# Supplementary material for: Effects of the Chloroplast Fructose-1,6-Bisphosphate Aldolase Gene on Growth and Low-Temperature Tolerance of Tomato
Source: Int J Mol Sci. 2022 Jan 10;23(2):728. doi: 10.3390/ijms23020728 (PMC8775715; doi:10.3390/ijms23020728)
Supplement: Supplementary file 1 [file ijms-23-00728-s001.zip › ijms-1502376-supplementary.pdf]

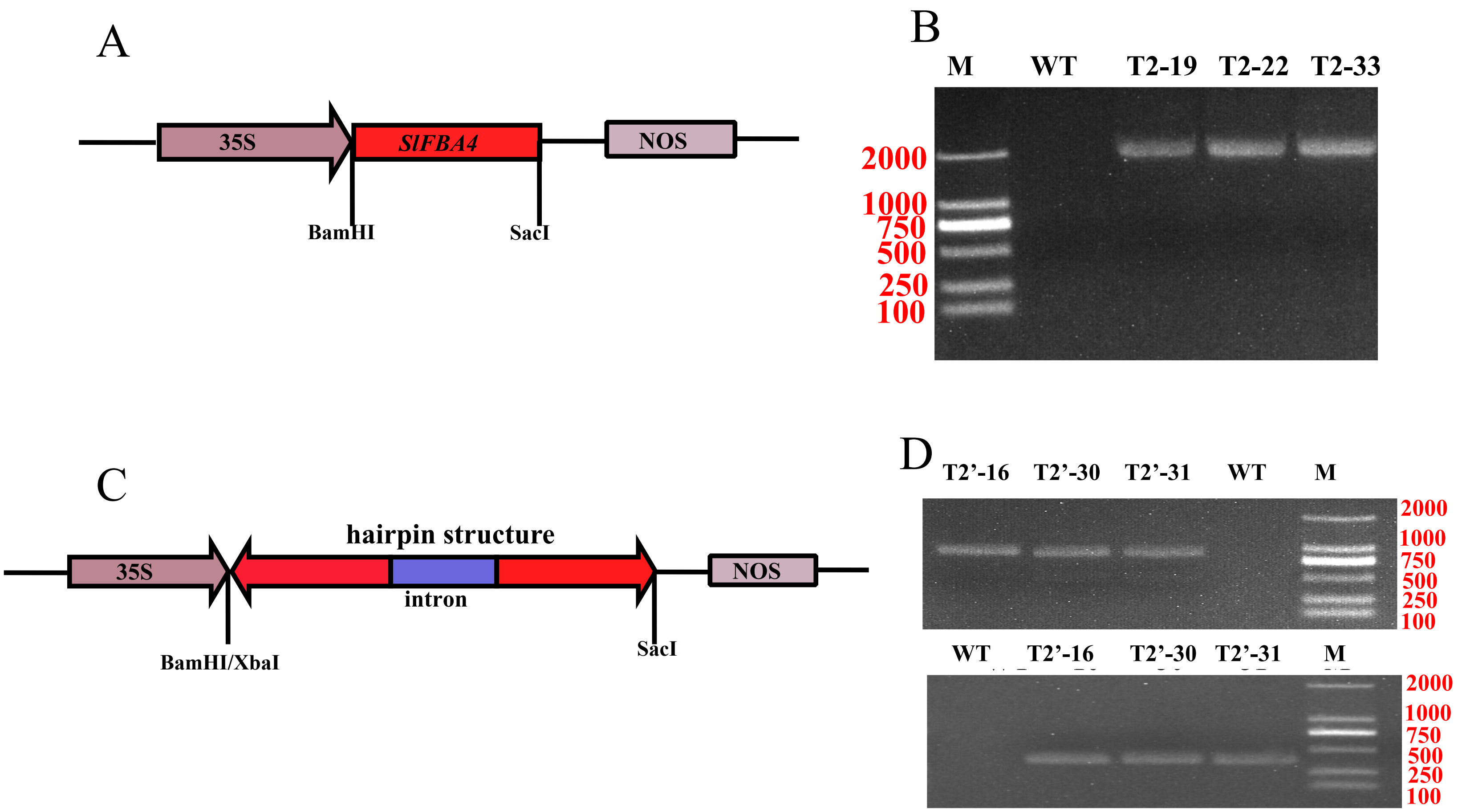

Figure S1. *SlFBA4* expression vector construction and PCR detection of transgenic plants. (**A**) Construction of *SlFBA4* overexpression vector. (**B**) The detection of overexpression transgenic tomato plants by PCR. M=DNA marker; WT= wild-type. (**C**) Construction of *SlFBA4* RNAi vector. (**D**) The detection of RNAi transgenic tomato plants by PCR. M=DNA marker; WT= wild-type.

Table S1. Primer Sequences for transgenic plants confirmation.

Primer Name	Primer Sequence （5’-3’）
35SF	GTCCCCAGATTAGCCTTT
FBA4R	GCGGTACCCTGGCTAACAAAGAGGAA
35SF	GACCAAAGGGCAATTGAGAC
RNAi-FBA4-R1	GCGTCGACTCTAGAGTGCTTAGTAGGTGTAGC
RNAi-FBA4-R2	GCGTCGACGAGCTCGTGCTTAGTAGGTGTAGC

Table S2. Primer Sequences for qPCR.

Primer Name	Primer Sequence (5’-3’)
SBP-QF	CGTGACATCTCCAACAGCTAAGG
SBP-QR	CATCGCTGCTGTAACCTCCAG
FBPase-QF	GAAGG AAACTACCAGCTC
FBPase-QR	GAGAACTCTTTGGTGACC
GAPDH-QF	CCAACTGTTGATGTGTCC
GAPDH-QR	CCACTCGTGTACTGTAAC
RbcL-QF	GCTCTGACCGAGATCTTT
RbcL-QR	ATCCAAAACGTCCACTGC
RbcS-QF	GAA CCATAAGTCACCAGG
RbcS-QR	AGTATCCTTCGGGCTTGT
TK-QF	GTGTCCTTCCATCATCTG
TK-QR	TATCAGCAGGAGCACTAG
actin-F	TGTTGC TATTCAGGCTGTGC
actin-R	CTGCTCCTGGCAGTTTCAAT
